# Sexual and reproductive health (SRH) needs of women admitted to eileen skellern ward (ES1) psychiatric intensive care unit (PICU)

**DOI:** 10.1192/j.eurpsy.2021.2155

**Published:** 2021-08-13

**Authors:** E. Rose, L. Blake, E. Covshoff, F. Sethi, R. Rathouse, A. Wilson, A. Bolade, R. Pittrof

**Affiliations:** 1 Psychiatric Intensive Care Unit, THE MAUDSLEY HOSPITAL, LONDON, United Kingdom; 2 Camberwell Sexual Health Clinic, KINGS COLLEGE LONDON, LONDON, United Kingdom

**Keywords:** PSYCHIATRIC INTENSIVE CARE UNIT, WOMEN’S MENTAL HEALTH, SEXUAL AND REPRODUCTIVE HEALTH, SEXUAL MEDICINE AND MENTAL HEALTH

## Abstract

**Introduction:**

PICU inpatients are likely to be at increased risk of having unmet SRH needs due to barriers to accessing services. Since May 2018, an in-reach SRH assessment has been available to all psychiatric inpatients on ES1 ward, if referred. Analysis of referrals over 15 months identified only 24 had been made during this time.

**Objectives:**

To assess the SRH needs of women admitted to ES1 PICU, the feasibility of providing a SRH in-reach clinic, and the acceptability of delivering a nurse lead referral programme.

**Methods:**

A bi-monthly SRH in-reach clinic and a nurse led SRH referral pathway were implemented on ES1 over a seven-month period. A staff training needs assessment was performed followed by training, a protocol was developed, staff attitudes were explored, and patient engagement was sought.

**Results:**

A total of 41% (32/77) of patients were referred, which was a 29% increase. 53.1% (17/32) of the total referrals had a true SRH need, equating to a 10% increase and 22% (17/77) of all PICU admissions. 90% of referrals were made by nursing staff. A staff focus group (n15) highlighted the acceptability and perceived importance of offering SRH care in PICU, if interventions were appropriately timed and the patient’s individual risk profile was considered.

**Conclusions:**

Results identify that SRH needs for PICU admissions are greater than previously realised. Providing a nurse led referral pathway for an SRH in-reach clinic is acceptable, feasible and beneficial for PICU patients. This project has resulted in service improvements including offering asymptomatic STI testing to all PICU admissions.

**Disclosure:**

No significant relationships.

